# Isolation and Characterization of New Anti-Inflammatory and Antioxidant Components from Deep Marine-Derived Fungus *Myrothecium* sp. Bzo-l062

**DOI:** 10.3390/md18120597

**Published:** 2020-11-26

**Authors:** Xiaojie Lu, Junjie He, Yanhua Wu, Na Du, Xiaofan Li, Jianhua Ju, Zhangli Hu, Kazuo Umezawa, Liyan Wang

**Affiliations:** 1 Shenzhen Key Laboratory of Marine Bioresource and Eco-environmental Science, College of Life Sciences and Oceanography, Shenzhen University, Shenzhen 518060, China; luxiaojie@szu.edu.cn (X.L.); hejunjie2017@email.szu.edu.cn (J.H.); duna2017@email.szu.edu.cn (N.D.); lixiaof@szu.edu.cn (X.L.); huzl@szu.edu.cn (Z.H.); 2 Key Laboratory of Optoelectronic Devices and Systems of Ministry of Education and Guangdong Province, College of Optoelectronic Engineering, Shenzhen University, Shenzhen 518060, China; 3 Department of Molecular Target Medicine, Aichi Medical University School of Medicine, Nagakute 480-1195, Japan; wu.yanhua.196@mail.aichi-med-u.ac.jp; 4 CAS Key Laboratory of Tropical Marine Bio-resources and Ecology, South China Sea Institute of Oceanology, Chinese Academy of Sciences, Guangzhou 510301, China; jju@scsio.ac.cn

**Keywords:** deep sea marine-derived fungus, *Myrothecium* sp., myrothecol, nitric oxide (NO), antioxidant activity

## Abstract

In the present study, four new compounds including a pair of 2-benzoyl tetrahydrofuran enantiomers, namely, (−)-1*S*-myrothecol (**1a**) and (+)-1*R*-myrothecol (**1b**), a methoxy-myrothecol racemate (**2**), and an azaphilone derivative, myrothin (**3**), were isolated along with four known compounds (**4**–**7**) from cultures of the deep-sea fungus *Myrothecium* sp. BZO-L062. Enantiomeric compounds **1a** and **1b** were separated through normal-phase chiral high-performance liquid chromatography. The absolute configurations of **1a**, **1b**, and **3** were assigned by ECD spectra. Among them, the new compound **1a** and its enantiomer **1b** exhibited anti-inflammatory activity, inhibited nitric oxide formation in lipopolysaccharide-treated RAW264.7 cells, and exhibited antioxidant activity in the 2,2-azino-bis(3-ethylbenzothiazoline-6-sulfonic acid) and oxygen radical absorbance capacity assays.

## 1. Introduction

Natural products are a rich source of new drugs and they are frequently used for the discovery and development of new drugs [1]. Natural marine products with unique architectures and distinct biological activities are treasure troves for natural product chemists [2,3]. Among marine organisms, fungi produce a diverse range of biologically active metabolites [3], including polyketides [4,5,6], terpenoids [7,8,9], polypeptides [10], and alkaloids [11,12,13].

Microorganisms of the deep-sea are an attractive source of candidate drugs. While screening inhibitors of lipopolysaccharide (LPS)-induced nitric oxide (NO) production, we recently isolated cyclopenol and cyclopenin from the extract of the fungal strain *Aspergillus* sp. SCSIOW2 collected from a depth of approximately 2000 m in the sea [14]. At non-toxic concentrations, these compounds inhibited LPS-induced NO production and IL-6 secretion in RAW264.7 cells. This inhibitory effect of cyclopenol and cyclopenin was attributed to the suppression of the upstream signal of NF-B activation. These compounds also suppressed the expression of IL-1β, IL-6, and iNOS in microglia cells (macrophages in the mouse brain) [14]. In Alzheimer’s disease, amyloid β-peptide induces inflammation in the brain. Between the two compounds, cyclopenin showed ameliorative effects in an in vivo Alzheimer’s model using flies [14].

**Figure 1:**
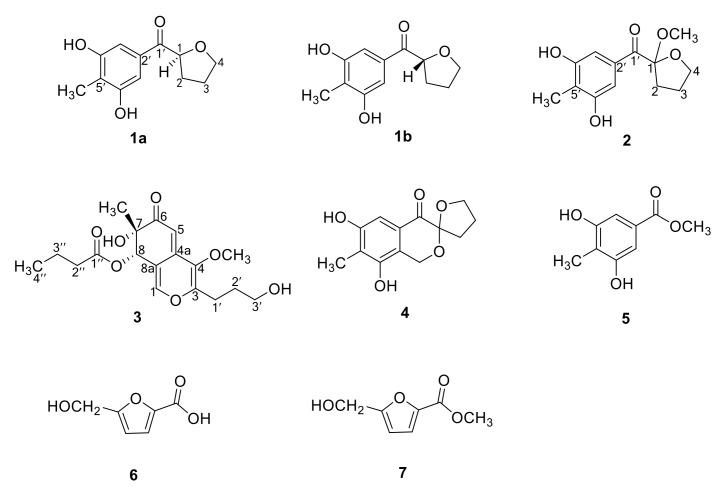
Compounds **1**–**7** isolated from *Myrothecium* sp. BZO-L062, including (−)-(1*S*)-myrotheciol (**1a**), (+)-(1*R*)-myrotheciol (**1b**), 1-methoxy-myrotheciol (**2**), myrothin (**3**), terreinol (**4**), 3,5-dihydroxy-4-methylbenzoic acid methyl ester (**5**), 5-hydroxymethyl-2-furoic acid (**6**), and 5-hydroxymethyl-2-furancarboxylic acid methyl ester (**7**).

To explore new bioactive secondary metabolites from deep marine-derived fungi [15,16,17], a fungal strain, *Myrothecium* sp. BZO-L062, isolated from sediment samples collected from the sea bottom near Yongxing Island, was used for chemical investigation. Seven pure components, including four new compounds (**1a**, **1b**, **2**, and **3**), were isolated and identified from the ethyl acetate extract of the fungus (Figure 1). The absolute configurations of the new compounds (**1a**, **1b**, and **3**) were assigned by comparison of their experimental CD spectra with the theoretically calculated spectra. The NO production inhibitory activity and antioxidant activity of the new compounds were also evaluated. Known compounds **4**–**7** were identified as terreinol (**4**) [18], 3,5-dihydroxy-4-methylbenzoic acid methyl ester (**5**), 5-hydroxymethyl-2-furoic acid (**6**) [19], and 5-hydroxymethyl-2-furancarboxylic acid methyl ester (**7**) [20] by comparing their spectroscopic data with those previously reported.

## 2. Results and Discussion

**Figure 2:**
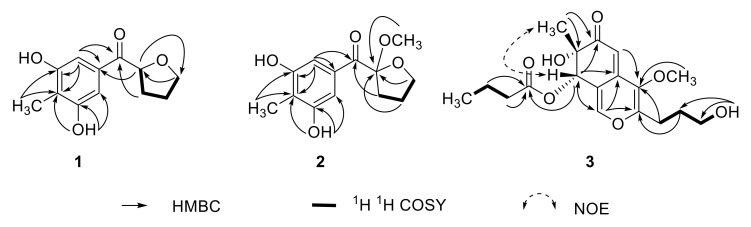
Key 2D NMR correlations of **1**–**3**.

The molecular formula of **1** was determined as C_12_H_14_O_4_ by high-resolution electrospray ionization mass spectrometry (HRESIMS) at *m*/*z* 223.0958 [M + H]^+^ and 245.0780 [M + Na]^+^ (calculated for C_12_H_15_O_4_^+^, 223.0965; C_12_H_14_O_4_Na^+^, 245.0784) (Figure S1). ^1^H NMR, ^13^C NMR, and 2D-NMR data of **1** (Table 1, Figures S2–S8) revealed the presence of 12 resonance signals, including those for one sp^3^ methyl, one sp^3^ oxygenated methine, three sp^3^ methylenes, two symmetric sp^2^ methines, two symmetric sp^2^ oxygenated quaternary carbons, two sp^2^ quaternary carbons, and one ketone carbonyl carbon. The ^1^H-^1^H correlation spectroscopy (COSY) data from H-1 to H_2_-4 and the ¹H-¹³C heteronuclear multiple bond correlations (HMBC) from H-1 to oxygenated C-4 and from H_2_-4 to C-1 suggested the presence of a tetrahydro-2-furanyl moiety (Table 1 and Figure 2). The four aromatic carbon signals indicated the presence of one symmetrically substituted benzene ring. The HMBC experiment correlations confirmed the presence of a 3,5-dihydroxy-4-methyl benzoyl moiety (Table 1 and Figure 2). Finally, the key HMBC correlations from H_2_-2 to C-1 and from H-1 to C-2 allowed the linkage of the 3,5-dihydroxy-4-methyl benzoyl and tetrahydro-2-furanyl groups (Table 1 and Figure 2). Accordingly, **1** was established as (3,5-dihydroxy-4-methylphenyl)-(tetrahydro-2-furanyl)methanone and denoted as a myrotheciol.

**Figure 3:**
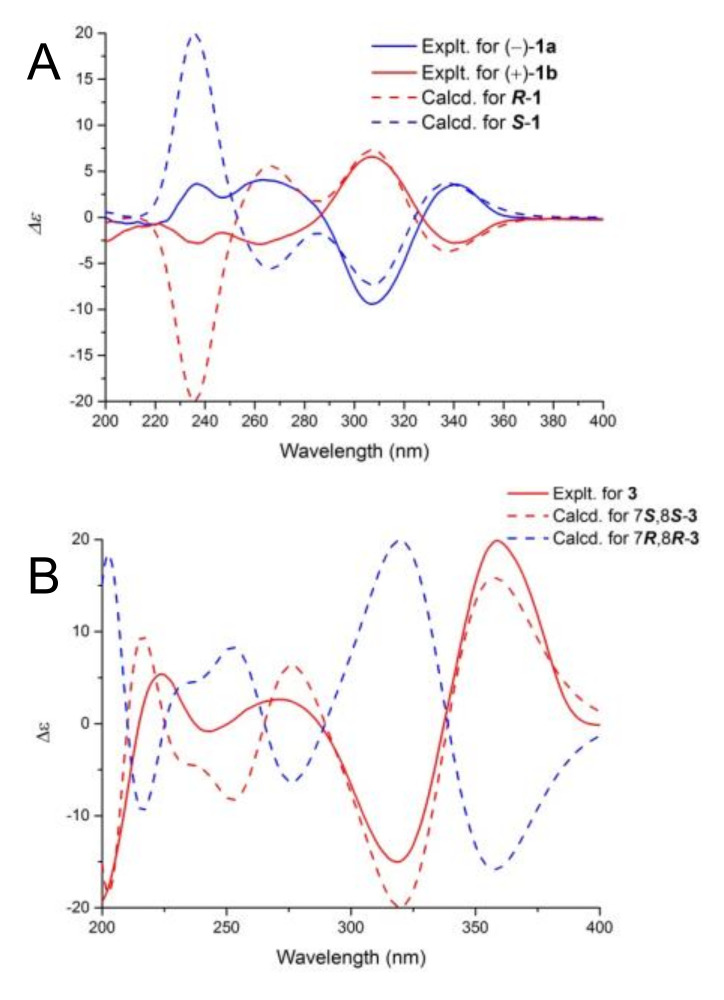
ECD spectra of compounds **1a** and 1**b** (**A**), and **3**(**B**).

The absence of the Cotton effect in the CD spectrum and zero specific rotation indicated that **1** was a racemate. Generally, enantiomers are more advantageous than racemates for drug development. To detect the enantiomers of **1**, chiral HPLC was performed using a Chiralpak IC column; the HPLC results showed two separate peaks (Figure S9). The two enantiomers, (−)-**1a** and (+)-**1b**, were obtained in a ratio of 1:1. (−)-**1a** and (+)-**1b** showed mirror image-like CD curves (Figure 3) and opposite specific rotations (**1a**: [α]$\begin{array}{c} 20\\ D \end{array}$ − 25.3; **1b**: [α]$\begin{array}{c} 20\\ D \end{array}$ + 26.3). The experimental CD spectra of **1a** were consistent with the theoretically calculated ECD spectrum of the **1-***S* enantiomer with four Cotton effects observed at 237 nm (positive), 270 nm (positive), 310 nm (negative), and 348 nm (positive) (Figure 3). In contrast, the CD spectrum of **1b** was consistent with the ECD spectrum of the 1-***R*** enantiomer but different from that of **1-***S* with three negative Cotton effects at 237 nm, 270 nm, and 348 nm, and one positive Cotton effect at 310 nm. Thus, the absolute configurations of **1a** and **1b** were assigned as (−)-(1*S*)-myrotheciol and (+)-(1*R*)-myrotheciol, respectively (Figure 1).

The molecular formula of **2** was determined as C_13_H_16_O_5_ through HRESIMS at *m*/*z* 275.0896 [M + Na]^+^ (calculated for C_13_H_16_O_5_Na^+^, 275.0890), which was 30 mass units larger than **1** (Figure S10). The ^1^H and ^13^C NMR data of **2** (Table 2, Figures S11–S16) closely resembled those of **1**, except for three major differences: the presence of an additional methoxy group (*δ*_H_ 3.09, *δ*_C_ 50.2), the absence of a methine proton (*δ*_H_ 5.09), and the chemical shift of C-1 (from *δ*_C_ 79.1 to 109.5); these differences indicated the substitution of the methine proton at C-1 by a methoxy group. The position of the new methoxy group was confirmed by HMBC correlation from 1-OMe to C-1 (Table 2 and Figure 2). Thus, **2** was established as 1-methoxy-myrotheciol. The structure of **2** was validated through a detailed analysis of 2D NMR data (Table 2 and Figure 2).

Compound **2** was also considered as a racemic mixture based on the zero specific rotation and absence of the Cotton effect in its CD spectrum. The chiral HPLC performed using the same condition as that used for **1** revealed two peaks, attributable to **2a** and **2b**, at a ratio of approximately 1:1 (Figure S17). However, due to the limited sample size, further isolation was not carried out.

(+)-HRESIMS at *m*/*z* 353.1599 [M + H]^+^ and 375.1418 [M + Na]^+^ (calculated for C_18_H_25_O_7_^+^, 353.1595; C_18_H_24_O_7_Na^+^, 375.1414) revealed the molecular formula of **3** as C_18_H_24_O_7_ (Figure S18). The 1D- and 2DNMR results revealed the presence of one sp^3^ oxygenated quaternary carbon, one sp^3^ oxygenated methine, two sp^2^ aromatic methines, four sp^2^ quaternary carbons, one ketone carbonyl carbon, one methoxy group, and one angular methyl group (Table 3 and Figures S19–25). Other than these signals, the ^1^H-^1^H COSY correlations from H-2″ to H-4″, along with the HMBC correlations from H-2″ and 3″ to C-1″ (Table 3 and Figure 2) indicated the presence of the butyl ester fragment. The ^1^H-^1^H COSY correlations from H-1 to 3-OH corresponded to the hydroxypropyl fragment (Table 3, Figure 2). The NMR data of the core structure of **3** closely resembled those of C-8 dihydro-azaphilone [21,22]. Careful HMBC analysis confirmed this structure (Table 3 and Figure 2). Finally, the key HMBC correlation from H-8 to C-1″connected the butyl ester side chain to C-8, that from H-1 and H-2 to C-3 connected the hydroxypropyl group moiety to C-3, and that from 4-OCH_3_ to C-4 connected the methoxy group to C-4 (Table 3 and Figure 2). Accordingly, **3** was established as myrothin (Figure 1).

The relative configuration of **3** at C-7 and C-8 was assigned by nuclear overhauser effect spectroscopy (NOESY) correlations. The strong NOESY correlation between 7-CH_3_ and H-8 indicated that 7-CH_3_ and H-8 occupied the same side of the ring (Figure 2 and Figure S25). Thus, the stereo-configurations of C-7 and C-8 are either *S*,*S* or *R*,*R*. The experimental ECD curve of **3** was consistent with that of the 7*S*, 8*S* epimer (Figure 3). The chiral carbons C-7 and C-8 were thus determined as 7*S* and 8*S*.

**Figure 4:**
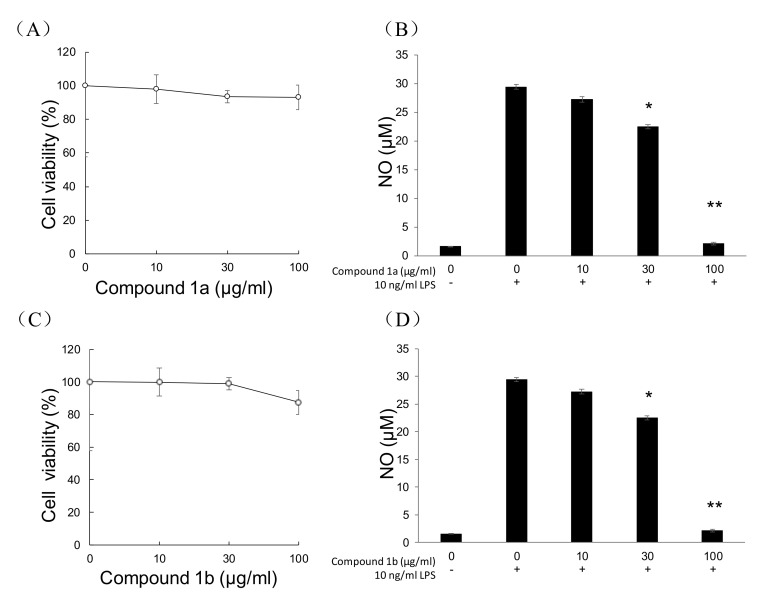
NO production inhibitory activity of **1a** and **1b** in RAW264.7 cells. Effect of **1a** (**A**) or **1b** (**C**) on the viability of RAW264.7 cells. Inhibition of LPS-induced NO production by **1a** (**B**) or **1b** (**D**). Values represent the means ± SEM of three independent experiments. *, *p* < 0.05; **, *p* < 0.001 vs. control.

LPS-induced NO production in RAW264.7 cells was used to evaluate the anti-inflammatory activity of different compounds [14]. NO is produced by NF-κB-dependent inducible NO synthase. All the isolated compounds were evaluated for cytotoxicity and for their effects on LPS-induced NO production. Among all the tested compounds, only two new compounds (**1a** and **1b**) significantly inhibited LPS-induced NO production at non-toxic concentrations (Figure 4).

Antioxidant activities were measured through 2,2-azino-bis(3-ethylbenzothiazoline-6-sulfonic acid) (ABTS) scavenging activity, 1,1-diphenyl-2-picrylhydrazyl (DPPH) scavenging capacity, and the oxygen radical absorbance capacity (ORAC) assay. As shown in Table 4, new compounds **1a** and **1b** exhibited antioxidant activity in the ABTS assay with EC_50_ of 1.20 and 1.41 µgmL^−1^, respectively, which were comparable with EC_50_ values of the positive controls L-ascorbic acid (1.55 µgmL^−1^) and trolox (1.61 µgmL^−1^). In the ORAC assay, the antioxidant ability was expressed as μmol trolox equivalents per μmol of sample solution. Compounds **1a** and **1b** showed high antioxidant activity (1.41 μM trolox/μM for **1a** and 1.19 μM trolox/μM for **1b**). Generally, the scavenging activities of ABTS are significantly higher than the scavenging activities of DPPH in phenolic compounds [23]. Compounds **1a** and **1b** did not show antioxidant activity in the DPPH assay, even at the highest concentration of 10 µgmL^−1^.

In the present research, we isolated several compounds including new structures from a deep-sea fungus. We found cellular anti-inflammatory activity in **1a** and **1b**. Microorganisms often produce useful compounds for therapy. However, the role of these compounds on producing organisms is not clear. At the beginning of antibiotic research, antibiotics are considered to protect the producing organisms by killing their enemy microorganisms. But later, many enzyme inhibitors such as pepstatin and leupeptin were discovered from the secondary metabolites of *Streptomyces*, and they showed no antibiotic activity. Therefore, it is unlikely that these secondary metabolites are useful for the producers. From this point of view, new compounds, **1a** and **1b**, may be remnants of microorganisms in their evolution.

## 3. Materials and Methods

### 3.1. General Experimental Procedures

Optical rotations were recorded on an Anton Paar MCP-100 polarimeter (Anton Paar GmbH, Graz, Austria). ECD spectra were measured on a JASCO-810 spectropolarimeter (JASCO Corporation, Tokyo, Japan). UV spectra were obtained on a UV-1800 spectrophotometer (Shimadzu Corporation, Tokyo, Japan). IR spectra were recorded on a Nicolet Avatar 330 FT-IR spectrometer (Thermo Scientific, Waltham, MA, USA) using KBr disks. NMR spectra were acquired on a Bruker ASCEND 500 MHz or 600 MHz NMR magnet system (Bruker, Ettlingen, Germany) using tetramethylsilane (TMS) as the internal standard. HRESIMS was performed using a Triple TOF 6600 (AB SCIEX LLC, Framingham, MA, USA). Column chromatography (CC) was conducted using silica gel (200–300 mesh, Qingdao Marine Chemical Factory, Qingdao, China) and Sephadex LH-20 (Amersham Pharmacia Biotech, Piscataway, NJ, USA). Thin-layer chromatography (TLC) was performed on Merck TLC plates silica gel 60 F_254_ and silica gel 60 RP-18 F_254S_ (Merck Millipore Corporation, Darmstadt, Germany). HPLC was carried out on a Shimadzu LC-16P HPLC system (Shimadzu Corporation, Tokyo, Japan) using YMC-pack Pro C18 Column (4.6 × 250 mm, 5 µm; 10 × 250 mm, 5 µm; YMC Co., Ltd., Kyoto, Japan) for analysis and semi-preparation. Optical pure compounds were prepared using a DAICEL Chiralpak IC column (250 mm × 4.6 mm, 5 µm; YMC Co., Ltd., Kyoto, Japan). All the chemical reagents for isolation were either of analytical (Damao Chemical Factory, Tianjin, China) or HPLC grade (Kermel Chemical Co., Ltd., Tianjin, China).

### 3.2. Fungal Material

The fungus *Myrothecium* sp. BZO-L062 used in this study was isolated from a deep-sea (2130 m depth) sediment sample collected from an area close to Yongxing Island, China. The strain was identified as *Myrothecium* sp. based on the morphological features and internal transcribed spacer sequence analysis. This strain was deposited at the Marine Natural Products Laboratory, College of Life Sciences and Oceanography, Shenzhen University, Shenzhen, China.

### 3.3. Fermentation and Extraction

The fungus *Myrothecium* sp. BZO-L062 was activated on petri dishes containing potato dextrose agar supplemented with 3% sea salt at 28 °C for three days [24]. Agar plugs were inoculated in a 500 mL Erlenmeyer flask containing 150 mL of liquid potato dextrose culture medium [24] supplemented with 3% sea salt as seed cultures and were incubated at 28 °C on a rotary shaker at 180 rpm for three days. Large-scale fermentation (70 L) was conducted using the same medium as that for seed cultures at 28 °C and 180 rpm for seven days. After seven days, the fermentation broth was filtered through cheesecloth to separate the supernatant from the mycelia. The supernatant was then concentrated to 8 L and successively extracted three times with EtOAc (3 × 8 L), yielding a crude extract (40.0 g).

### 3.4. Isolation and Purification

The crude extract was separated using silica gel CC through CH_2_Cl_2_/MeOH gradient elution (100:0, 100:1, 100:5, 100:10, 100:20, 100:50, and 0:100; 600 mL each) and was grouped into nine fractions (Fr.) based on the TLC analysis (Fr.1 to Fr.9). Fr.3 was purified by semi-preparative HPLC (28% MeCN/H_2_O, flow rate 3 mLmin⁻^1^) to yield **4** (t_R_ 16.2 min, 10.1 mg). Fr.4 was subjected to HPLC using a medium-pressure octadecyl-silica (ODS) column and separated with MeOH/H_2_O (20–100%) into five fractions (Fr.4.1–Fr.4.5). Fr.4.1 was further fractionated by HPLC (5% MeOH/H_2_O, a flow rate of 3 mLmin⁻^1^) to obtain **6** (t_R_ 15.0 min, 5.0 mg) and **7** (t_R_ 24.0 min, 5.0 mg). Fr.4.2 was purified by HPLC (25% MeOH/H_2_O, a flow rate of 3 mLmin⁻^1^) to obtain **3** (t_R_ 21.0 min, 1.0 mg). Fr.4.3 was refined by HPLC (25% MeCN/H_2_O, a flow rate of 3 mLmin⁻^1^) to obtain **2** (t_R_ 20.0 min, 1.4 mg). Finally, Fr.5 was subjected to HPLC (17% MeCN/H_2_O, flow rate 3 mLmin⁻^1^) to obtain **1** (t_R_ 20.0 min, 14.2 mg) and **5** (t_R_ 21.2 min, 10.2 mg).

The racemic compound **1** was resolved into enantiomers (−)-**1a** (3.0 mg, *t*_R_ 10.2 min) and (+)-**1b** (3.6 mg, *t*_R_ 18.1 min) using a chiral HPLC equipped with a DAICEL^®^ Cellulose Chiralpak IC column (5 µm, 4.6 × 250 mm) using *n*-hexane-ethanol (89:11) as mobile phase at a flow rate of 1 mLmin^−1^.

### 3.5. Spectral Data of the Compounds

#### 3.5.1. (±)-Myrothecol (1)

Myrothecol (**1**) is a colorless oil; [α]$\begin{array}{c} 20\\ D \end{array}$ 0°(*c* 0.1, MeOH); UV (MeOH) λ_max_ (log ε) 280 nm (7.18) and 218 nm (7.46); IR (KBr) ν_max_ 3325, 2956, 1678, 1591, 1423, 1325, 1198, 1088, 1040, 934, and 851; HRESIMS *m/z* 223.0958 [M+H]^+^, 245.0780 [M+Na]^+^ (calculated for C_12_H_15_O_4_, 223.0965; C_12_H_14_O_4_Na, 245.0784); for ^1^H NMR (DMSO-*d*_6_, 600 MHz) and ^13^C NMR (DMSO-*d*_6_, 150 MHz) spectral data, see Table 1. (−)-**1a**: [α]$\begin{array}{c} 20\\ D \end{array}$—25.3° (*c* 0.3, MeOH); ECD (2.3 mM, MeOH) λ_max_ (Δε) 237 nm (+0.49), 262 nm (+0.54), 307 nm (−1.27), 341 nm (+0.48). (+)-**1b**: [α]$\begin{array}{c} 20\\ D \end{array}$ + 26.3° (*c* 0.27, MeOH); ECD (2.3 mM, MeOH) λ_max_ (Δε) 237 nm (−0.38), 262 nm (−0.39), 307 nm (+0.88), and 341 nm (−0.37).

#### 3.5.2. Methoxy-myrothecol (2)

Methoxy-myrothecol (2) is a colorless oil; [α]$\begin{array}{c} 20\\ D \end{array}$ 0° (*c* 0.1, MeCN); UV (MeOH) λ_max_(log ε) 285 nm (6.99) and 218 nm (7.24); HRESIMS *m/z* 275.0896 [M+Na]^+^ (calculated for C_13_H_16_O_5_Na, 275.0890); HRESIMS *m/z* 353.1599 [M+H]^+^, 375.1418 [M+Na]^+^ (calculated for C_18_H_25_O_7_, 353.1595; C_18_H_24_O_7_Na, 375.1414); for _¬_^1^H NMR (DMSO-*d*_6_, 600 MHz) and ^13^C NMR (DMSO-*d*_6_, 150 MHz) spectral data, see Table 2.

#### 3.5.3. Myrothin (3)

Myrothin (**3**) is a light-yellow colored oil; UV (MeOH) λ_max_ (log ε) 246 nm (3.13) and 350 nm (3.56); IR (KBr) ν_max_ 3405, 2925, 2376, 2316, 1621, 1385, 1036, 910, 790, 731, and 635 cm⁻^1^; HRESIMS m/z 353.1599 [M+H]^+^, 375.1418 [M+Na]^+^, 727.2948 [2M+Na]^+^ (calculated for C_18_H_25_O_7_, 353.1595; C_18_H_24_O_7_Na, 375.1414; C_36_H_48_O_14_Na, 727.2936); for _¬_^1^H NMR (DMSO-*d*_6_, 600 MHz) and ^13^C NMR (DMSO-*d*_6_, 150 MHz) spectral data, see Table 3. [α]$\begin{array}{c} 20\\ D \end{array}$ + 15.7°; ECD (2.8 mM, MeOH) λ_max_ (Δε) 224 nm (+0.7), 243 nm (−0.11), 271 nm (+0.34), 319 nm (+1.96), and 359 nm (+2.59).

### 3.6. ECD Calculation

The conformational distribution search was conducted with the MMFF94 molecular mechanics force field in Spartan 12 software (Wavefunction Inc., Irvine, CA, USA). The lowest energy conformers within the 5-kcalmol^−1^ energy window were optimized using the Gaussian 09 program [25]. TDDFT calculations for all optimized conformers were performed at the B3LYP/6-31G (d, p) level. The ECD spectra were generated using the software SpecDis [26].

### 3.7. MTT and NO Production Assay

MTT and NO production inhibitory activities of the isolated compounds in RAW264.7 cells were determined as reported previously [14].

### 3.8. Antioxidant Activity

The ABTS and DPPH scavenging assays were carried out as reported earlier [23]. L-ascorbic acid and trolox were used as positive controls. The ORAC assay was conducted according to a previously reported protocol [27]. The results were expressed as μmol Trolox equivalents per μmol of sample solution.

## 4. Conclusions

In this study, four new components, (−)-1S-myrothecol (**1a**), (+)-1R-myrothecol (**1b**), methoxy-myrothecol (**2**), and myrothin (**3**), along with four known compounds (**4**–**7**), were isolated from the deep-sea fungus *Myrothecium* sp. BZO-L062. The enantiomers **1a** and **1b** were purified by chiral HPLC. The absolute configurations of **1a**, **1b**, and **3** were determined by the calculated ECD.

Among these compounds, new compounds **1a** and **1b** showed anti-inflammatory and antioxidant activities at non-toxic concentrations. Derivatives of these compounds could be potent and safe and may be useful for the development of new anti-inflammatory agents.

## Figures and Tables

**Table 1:** ^1^H NMR (600 MHz) and ^13^C NMR (150 MHz) spectral data of **1**.

No.	*δ*_C_	*δ*_H_, Mult. (*J* in Hz)	^1^H-^1^H COSY	HMBC
1	79.1	5.09, dd (8.4, 5.6)	2	C-2,3,4
2	29.0	2.17, m; 1.92, m	1,3	C-1,3,4,1′
3	25.2	1.84, m	2,4	C-1,2,4
4	68.4	3.81, t (6.7)	3	C-1,2,3
1′	198.0	-		
2′	132.7	-		
3′,7′	106.1	6.92, s		C-1′,2′,4′(6′),5′
4′,6′	156.1	-		
5′	116.7	-		
4′−OH/6′−OH	-	9.51, s		C-3′,4′,5′/C-5′,6′,7′
5′−CH_3_	8.9	1.99, s		C-4′,5′,6′

**Table 2:** ^1^H NMR (600 MHz) and ^13^C NMR (150 MHz) spectral data of **2**.

No.	*δ*_C_	*δ*_H_, Mult. (*J* in Hz)	^1^H-^1^H COSY	HMBC
1	109.5	-		
2	34.6	2.13, m	3	C-1,3,4,1′
3	24.0	1.88, m; 2.01, m	2,4	C-1,2,4
4	68.1	3.96, m	3	C-1,2,3
1′	194.8	-		
2′	131.7	-		
3′,7′	107.3	7.09, s		C-1′,2′,4′(6′),5′
4′,6′	155.9	-		
5′	116.7	-		
1−OCH_3_	50.2	3.09, s		C-1
4′−OH/6′−OH	-	9.47, s		C-3′,4′,5′/ C-5′,6′,7′
5′−CH_3_	8.9	1.98, s		C-4′,5′,6′

**Table 3:** ^1^H NMR (600 MHz) and ^13^C NMR (150 MHz) data of **3**.

No.	*δ*_C_	*δ*_H_, Mult. (*J* in Hz)	^1^H-^1^H COSY	HMBC
1	146.9	7.66, d (1.2)		C-3,4a,8,8a
3	154.7	-		
4	138.3	-		
4a	139.6	-		
5	99.7	5.28, d (1.2)		C-4,7,8a
6	196.5	-		
7	73.29	-		
8	73.30	5.54, s		C-1,4a,6,7,8a,1″
8a	116.5	-		
1′	24.2	2.58, m	2′	C-3,4,2′,3′
2′	29.6	1.68, m	1′,3′	C-3,1′,3′
3′	59.88	3.44, m	2′,3′-OH	C-1′,2′
3′-OH	-	4.58, t (5.1)	3′	C-2′,3′
4-OCH_3_	59.94	3.62, s		C-4
7-CH_3_	23.4	1.16, s		C-6,7
7-OH	-	5.07, s		C-6,7,7-CH_3_
1″	172.2	-		
2″	35.4	2.26, t (7.2)	3″	C-1″,3″,4″
3″	17.9	1.49, m	2″, 4″	C-1″,2″,4″
4″	13.2	0.82, t (7.4)	3″	C-2″,3″

**Table 4:** Antioxidant activities of **1a** and **1b**.

Compounds	ABTS	ORAC
	EC_50_, μg/mL	μM Trolox Equivalent/μM
1a	1.20 ± 0.18	1.41 ± 0.27
1b	1.41 ± 0.19	1.19 ± 0.19
*L*-Ascorbic acid	1.55 ± 0.15	0.35 ± 0.14
Trolox	1.61 ± 0.09	NA

## Supplementary Materials

The following are available online at https://www.mdpi.com/1660-3397/18/12/597/s1, Figure S1: HR-ESI MS spectrum of compound **1**, Figure S2–S8: 1D and 2D NMR spectra of compound **1**, Figure S9: Chiral separation of racemic 1, Figure S10: HR-ESI MS spectrum of compound 2, Figure S11–16: 1D and 2D NMR spectra of compound 2, Figure S17: Chiral separation of racemic 2, Figure S18: HR-ESI MS spectrum of compound 3, Figure S19–S25: 1D and 2D NMR spectra of compound **3**.
