# Supplementary material for: Isolation and Characterization of New Anti-Inflammatory and Antioxidant Components from Deep Marine-Derived Fungus Myrothecium sp. Bzo-l062
Source: Mar Drugs. 2020 Nov 26;18(12):597. doi: 10.3390/md18120597 (PMC7760613; doi:10.3390/md18120597)
Supplement: Supplementary file 1 [file marinedrugs-18-00597-s001.pdf]

Isolation and Characterization of New Anti-Inflammatory and Antioxidant Components from Deep Marine-Derived Fungus *Myrothecium* SP. Bzo-l062

Xiaojie Lu [1,2,†], Junjie He [1,†], Yanhua Wu [3], Na Du [1], Xiaofan Li [1], Jianhua Ju [4], Zhangli Hu [1,2], Kazuo Umezawa [3,*] and Liyan Wang [1,*]

[1] Shenzhen Key Laboratory of Marine Bioresource and Eco-environmental Science, College of Life Sciences and Oceanography, Shenzhen University, Shenzhen 518060, China; luxiaojie@szu.edu.cn (X.L.); hejunjie2017@email.szu.edu.cn (J.H.); duna2017@email.szu.edu.cn (N.D.); lixiaof@szu.edu.cn (X.L.); huzl@szu.edu.cn (Z.H.)

[2] Key Laboratory of Optoelectronic Devices and Systems of Ministry of Education and Guangdong Province, College of Optoelectronic Engineering, Shenzhen University, Shenzhen 518060, China

[3] Department of Molecular Target Medicine, Aichi Medical University School of Medicine, Nagakute 480-1195, Japan; wu.yanhua.196@mail.aichi-med-u.ac.jp

[4] CAS Key Laboratory of Tropical Marine Bio-resources and Ecology, South China Sea Institute of Oceanology, Chinese Academy of Sciences, Guangzhou, Guangdong 510301, China; jju@scsio.ac.cn

* Correspondence: lwang@szu.edu.cn (L.W.); umezawa@aichi-med-u.ac.jp (K.U.); Tel.: +86-755-2601-2653 (L.W.); +81-561-61-1959 (K.U.)

† These authors contributed equally to this work.

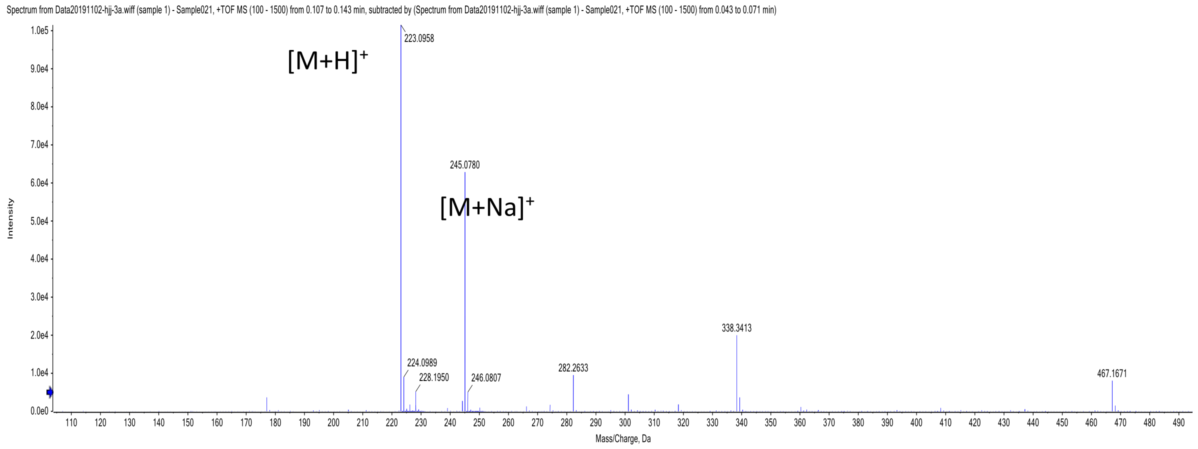

Figure S1. HR-ESI MS spectrum of compound **1**

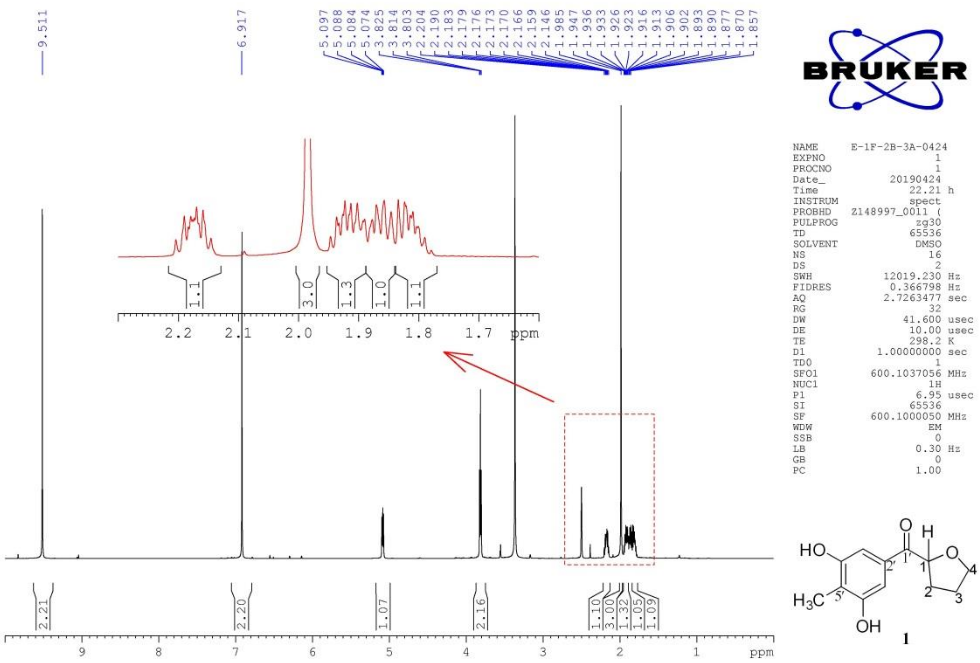

Figure S2. ^{1}H NMR spectrum of compound **1** in DMSO-d_6

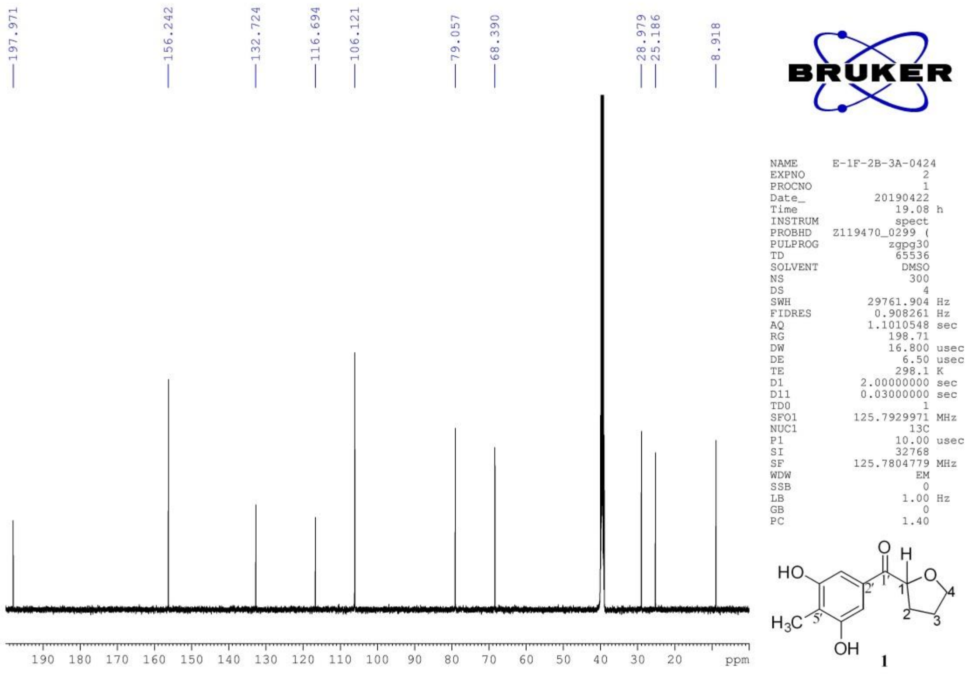

Figure S3. ^{13}C NMR spectrum of compound **1** in DMSO-d_6

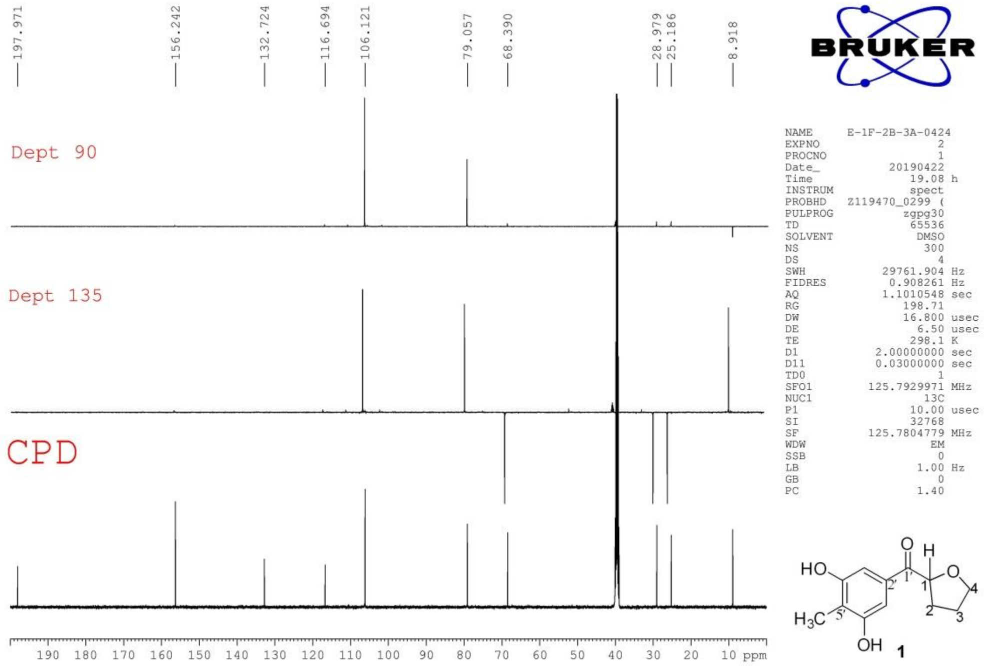

Figure S4. DEPT spectrum of compound **1** in DMSO-d_6

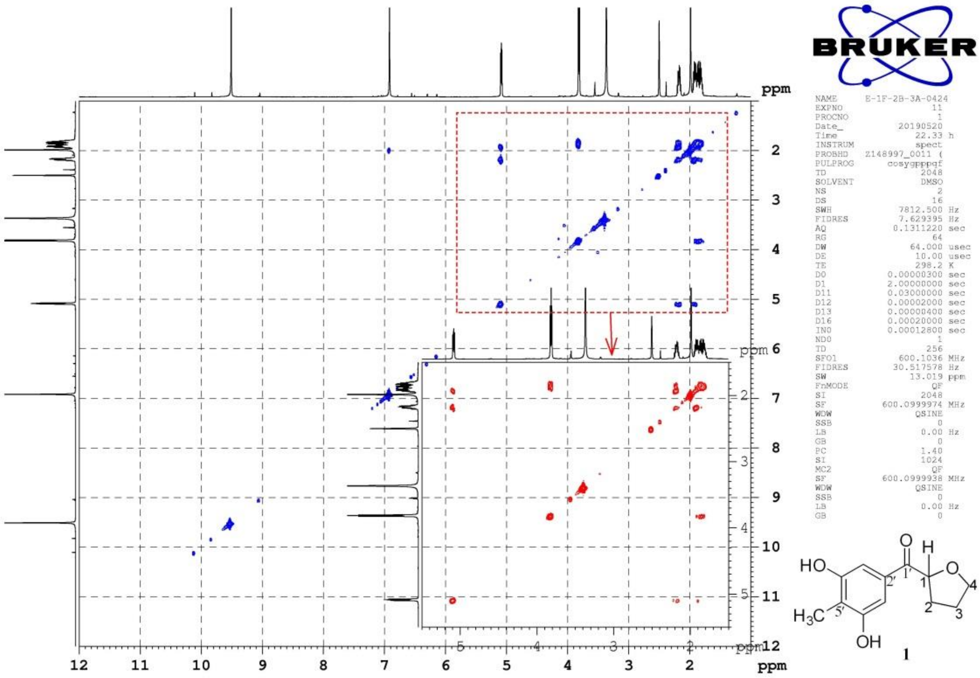

Figure S5 ^1H-^1H COSY spectrum of compound **1** in DMSO-d_6

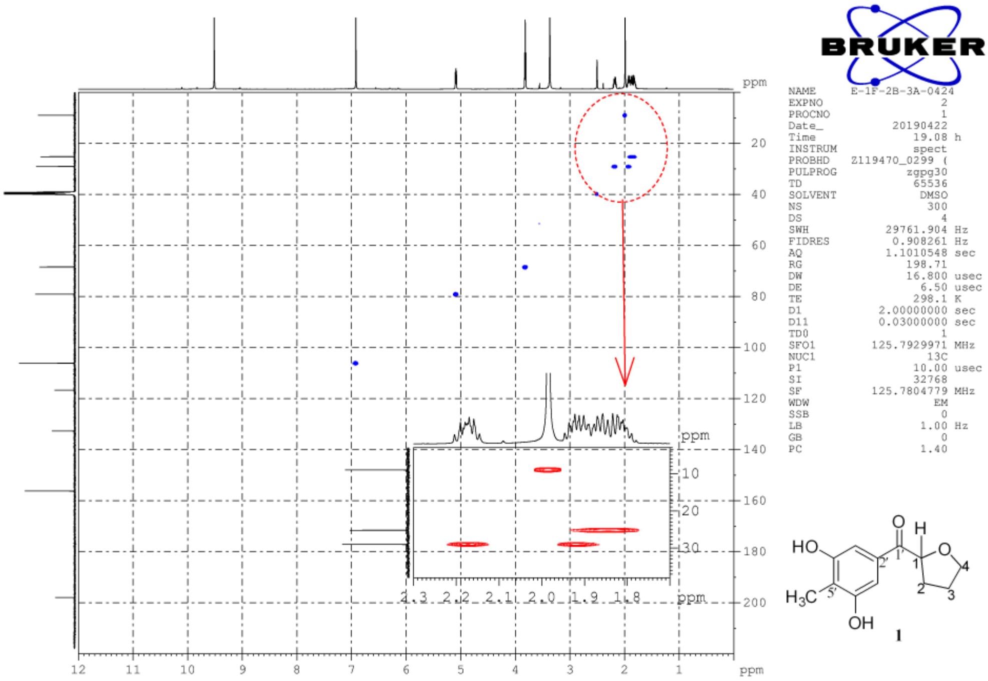

Figure S6. HSQC spectrum of compound **1** in DMSO-d_6

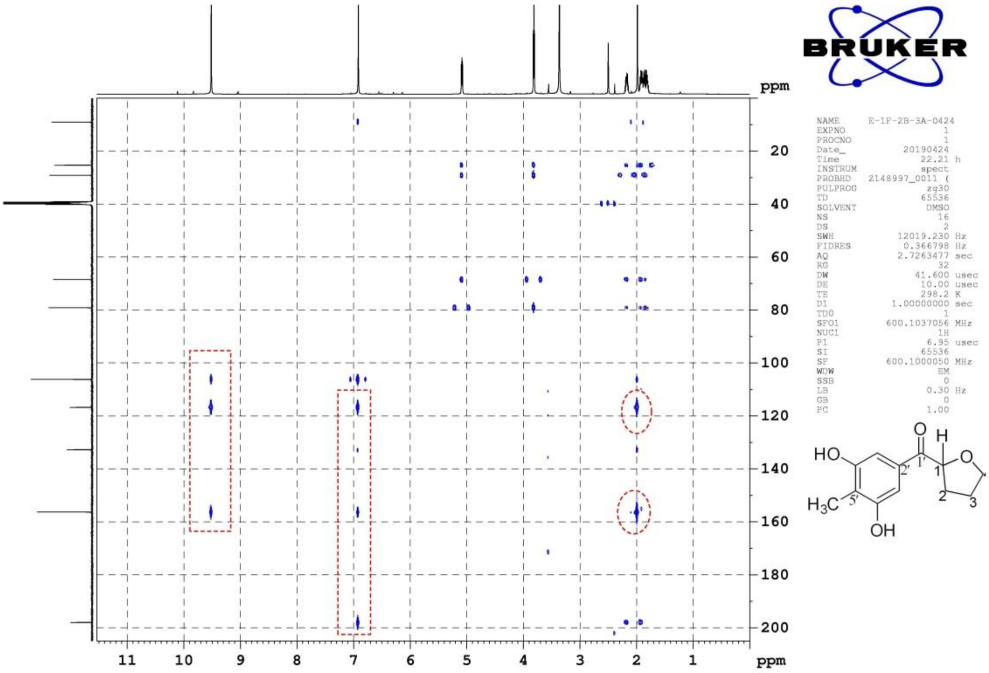

Figure S7 HMBC spectrum of compound **1** in DMSO-d_6

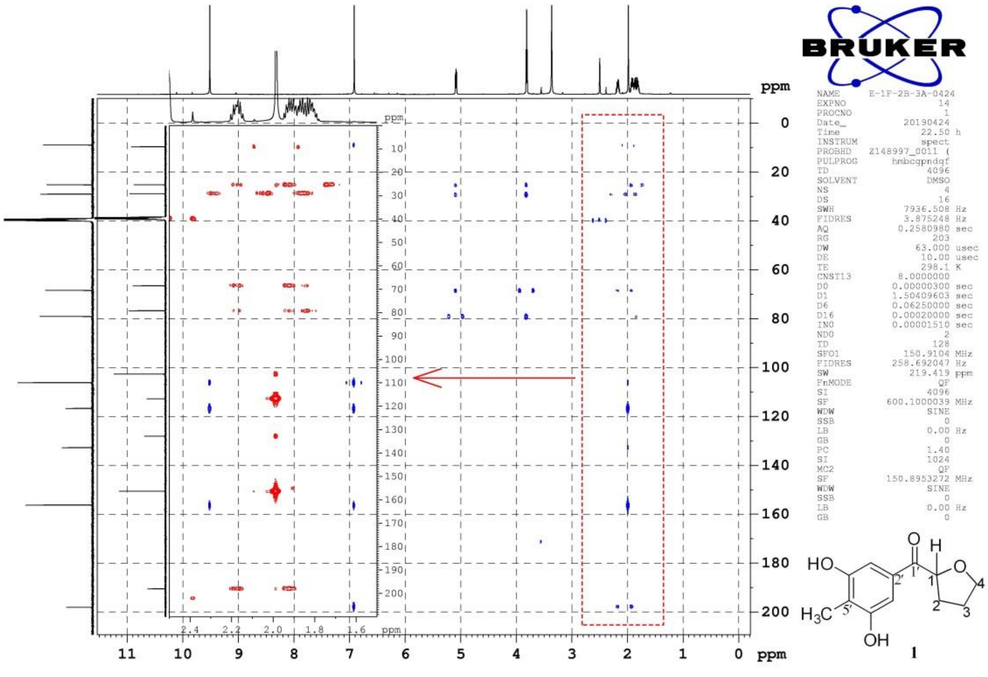

Figure S8 Partial HMBC spectrum of compound **1** in DMSO-d_6

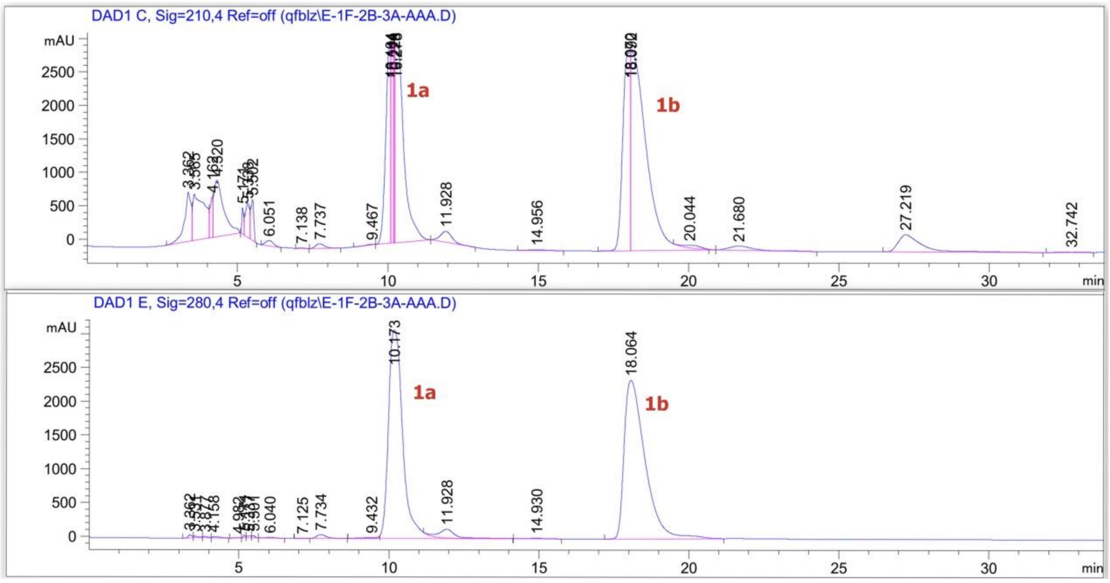

Figure S9 Chiral separation of racemic **1**

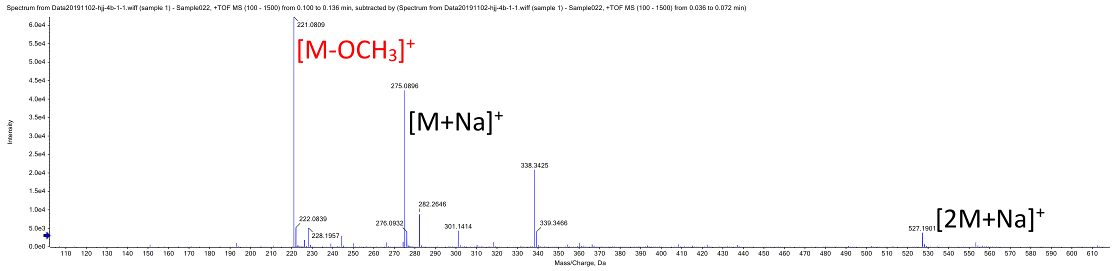

Figure S10. HR-ESI MS spectrum of compound **2**

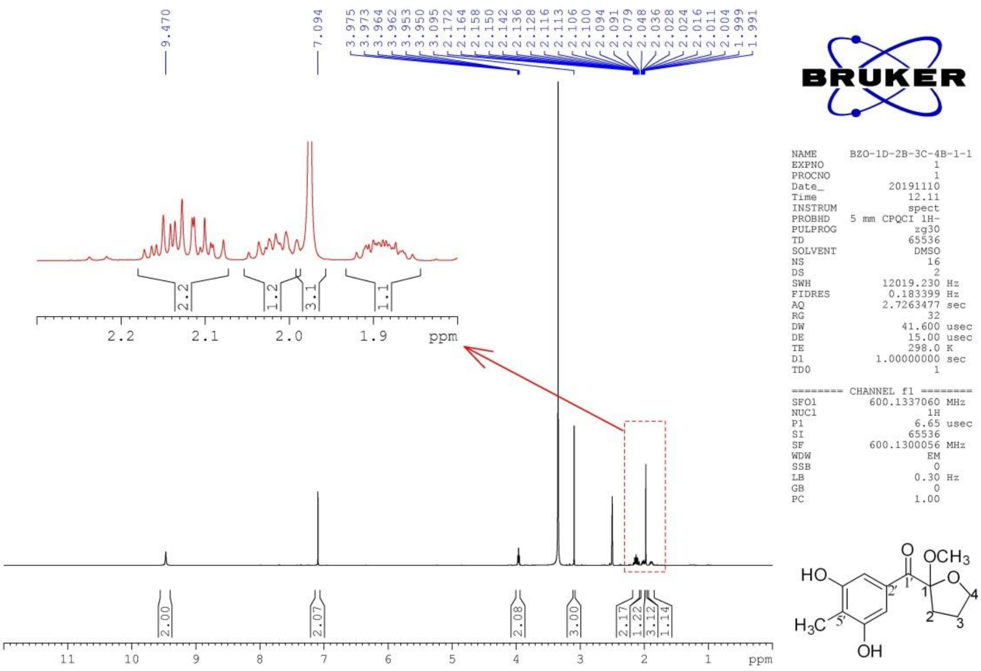

Figure S11. ^{1}H NMR spectrum of compound **2** in DMSO-d_6

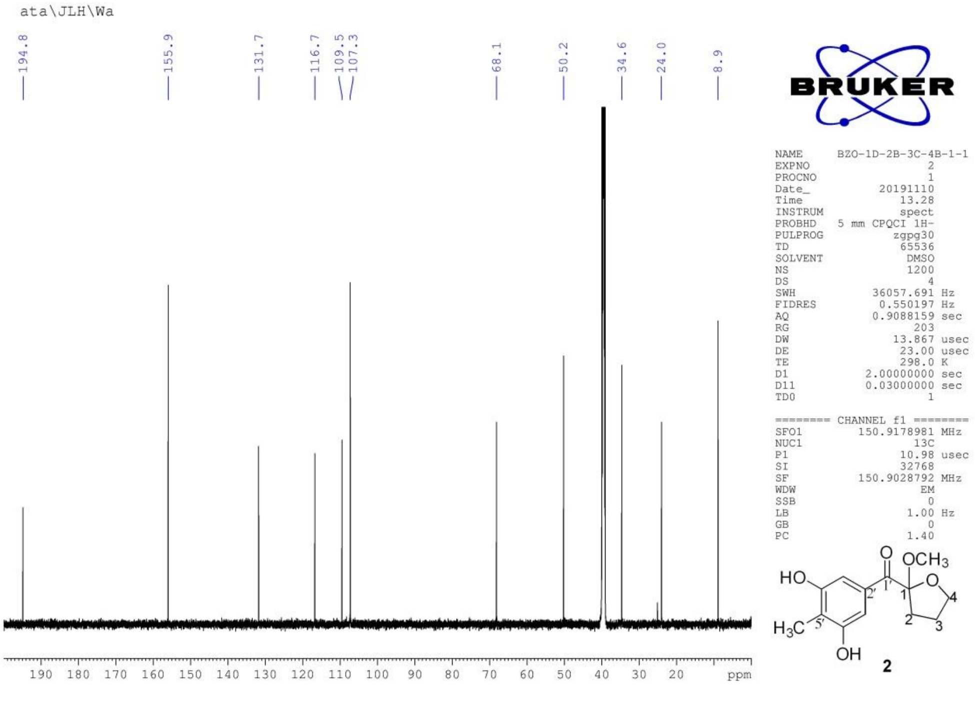

Figure S12. ^{13}C NMR spectrum of compound **2** in DMSO-d_6

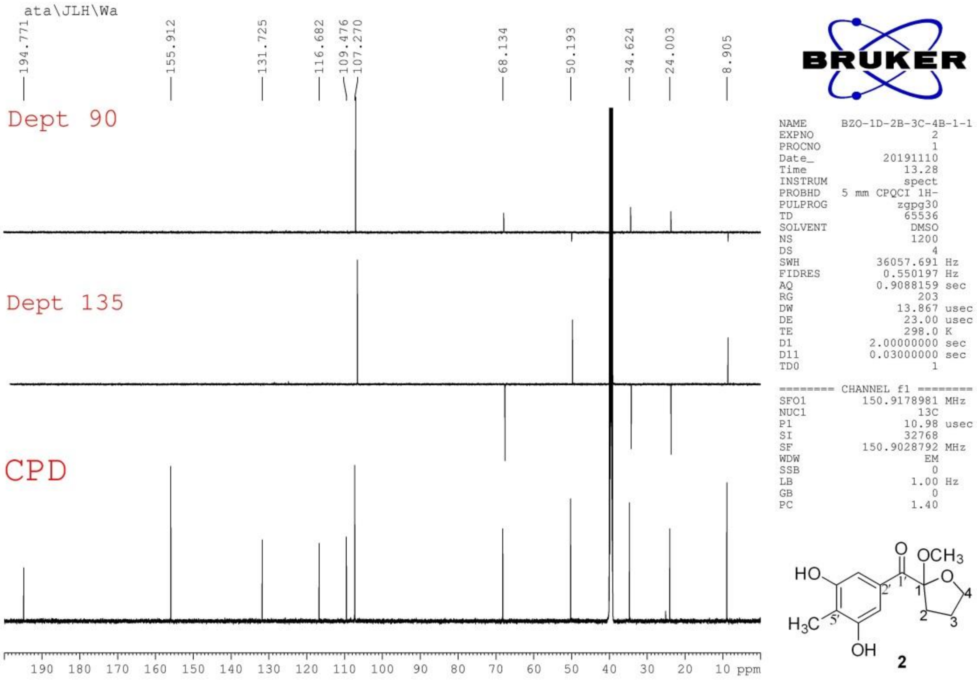

Figure S13. DEPT spectrum of compound **2** in DMSO-d_6

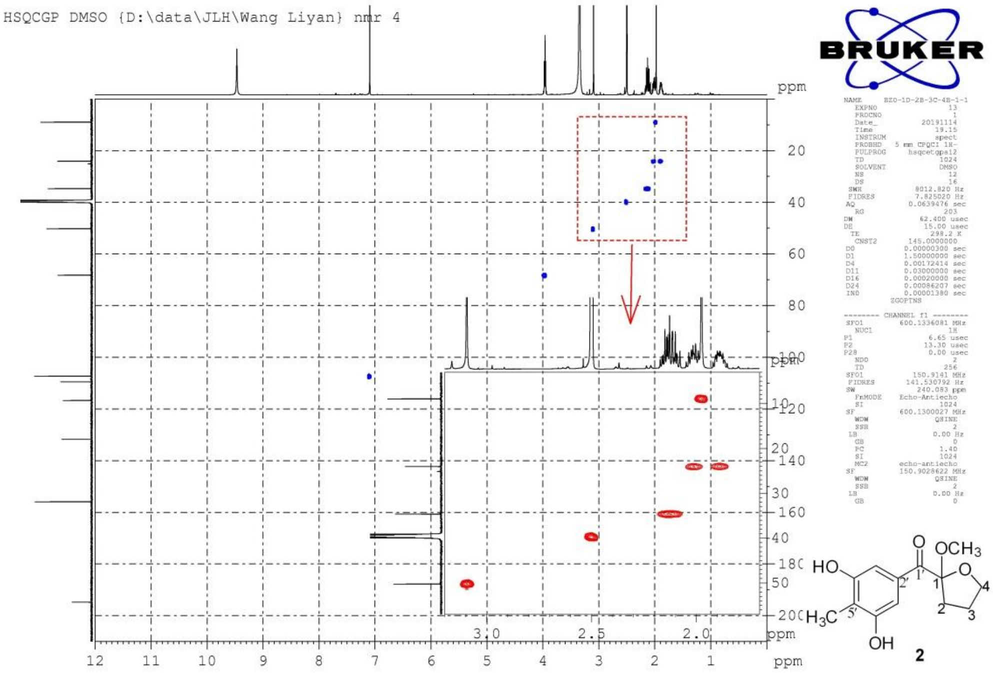

Figure S14. HSQC spectrum of compound **2** in DMSO-d_6

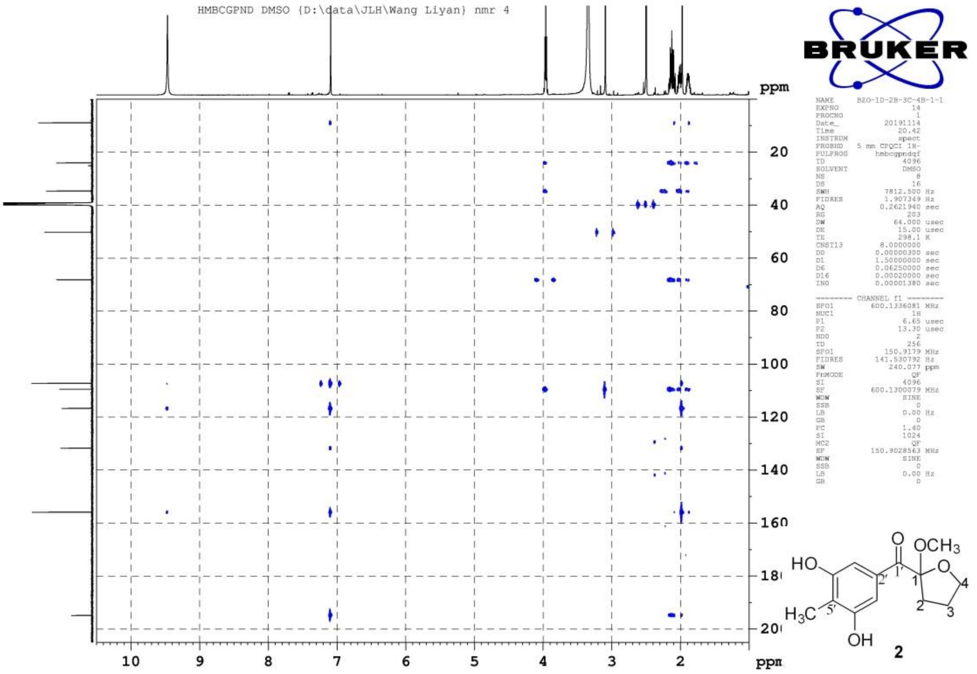

Figure S15. The HMBC spectrum of compound **2** in DMSO-d_6

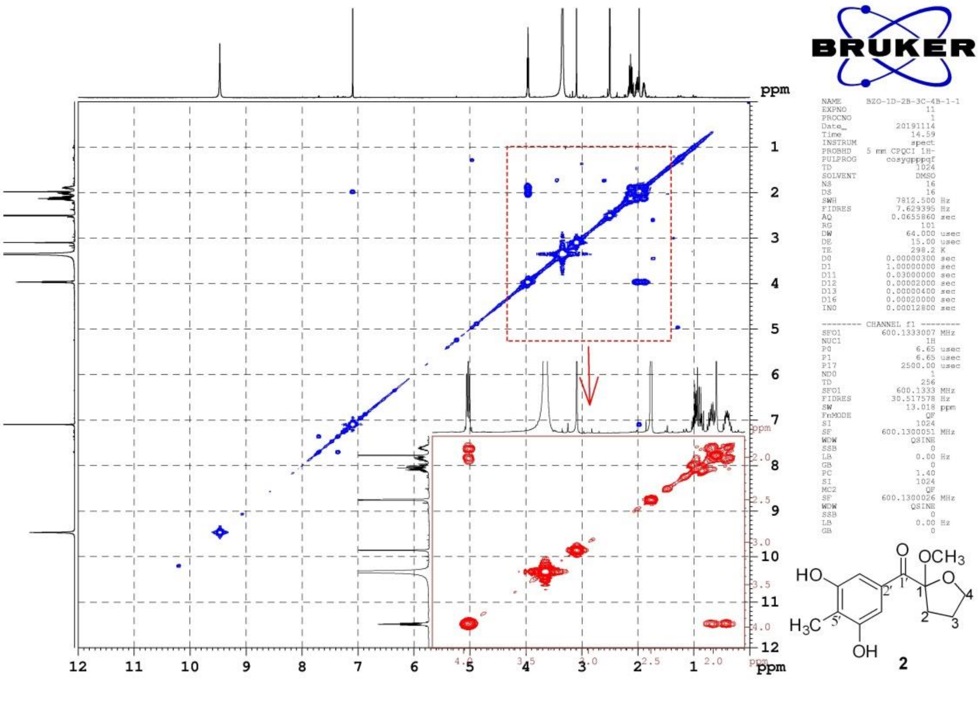

Figure S16. The ^{1}H-^{1}H COSY spectrum of compound **2** in DMSO-d_6

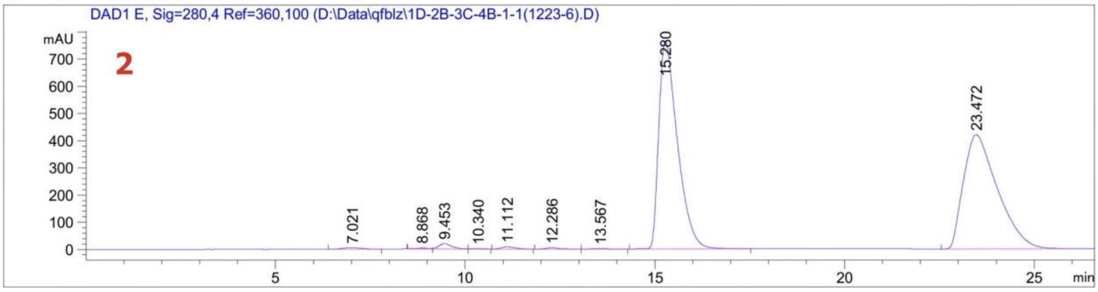

Figure S17. Chiral separation of racemic **2**

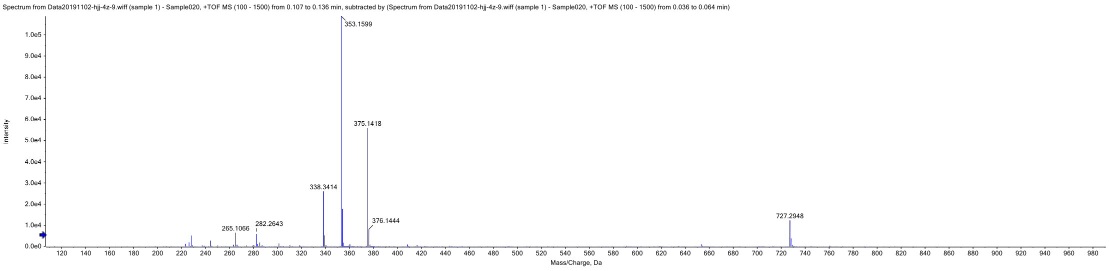

Figure S18. HR-ESI MS spectrum of compound **3**

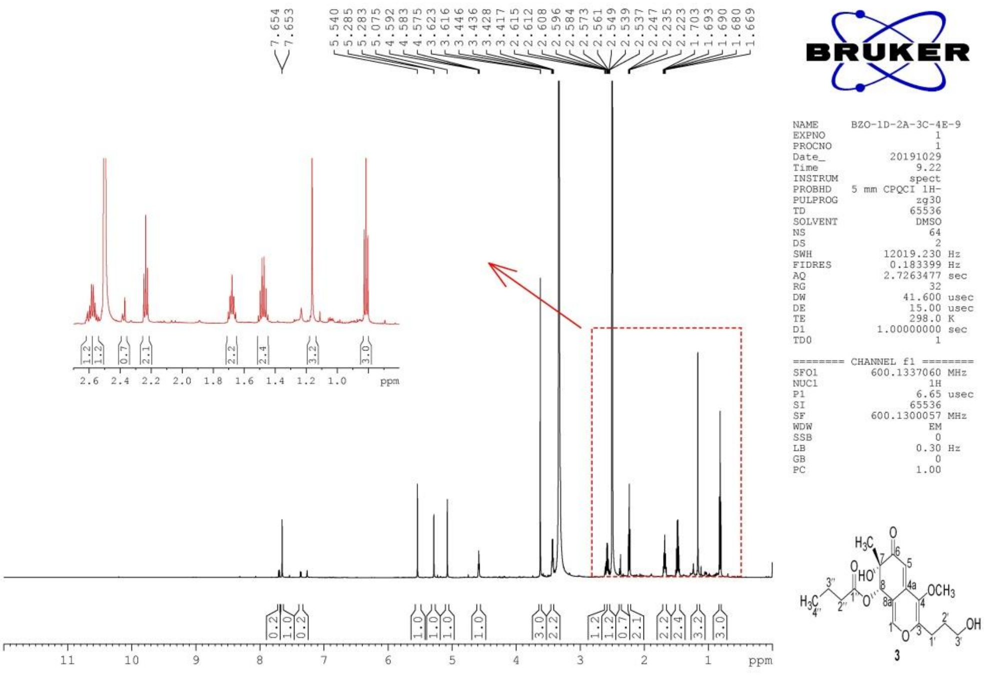

Figure S19. ^{1}H NMR spectrum of compound **3** in DMSO-d_6

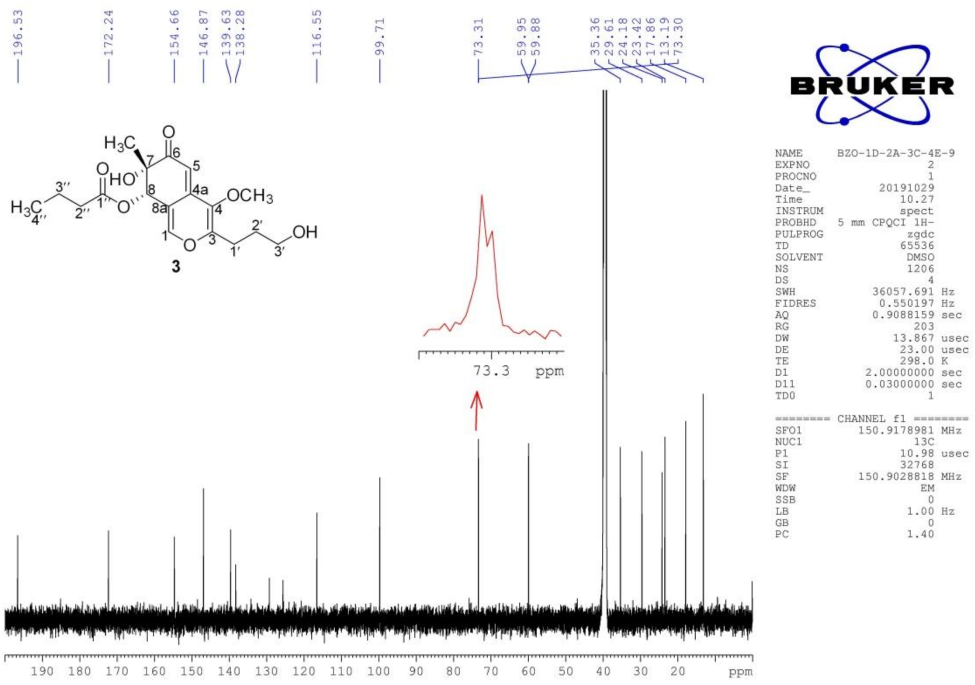

Figure S20. ^{13}C NMR spectrum of compound **3** in DMSO-d_6

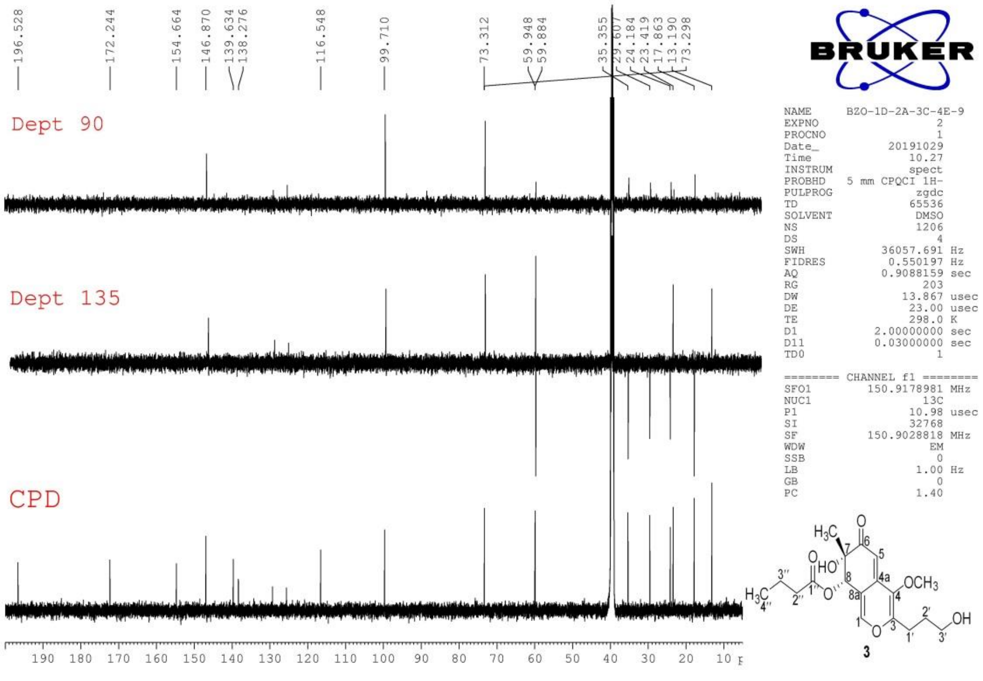

Figure S21. DEPT spectrum of compound **3** in DMSO-d_6

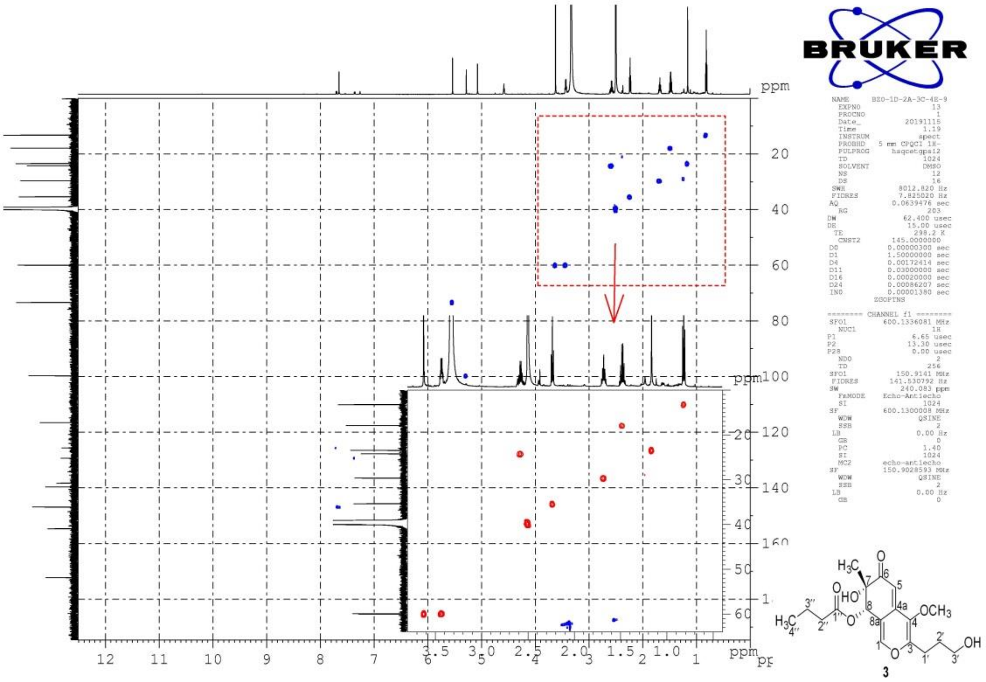

Figure S22. HSQC spectrum of compound **3** in DMSO-d_6

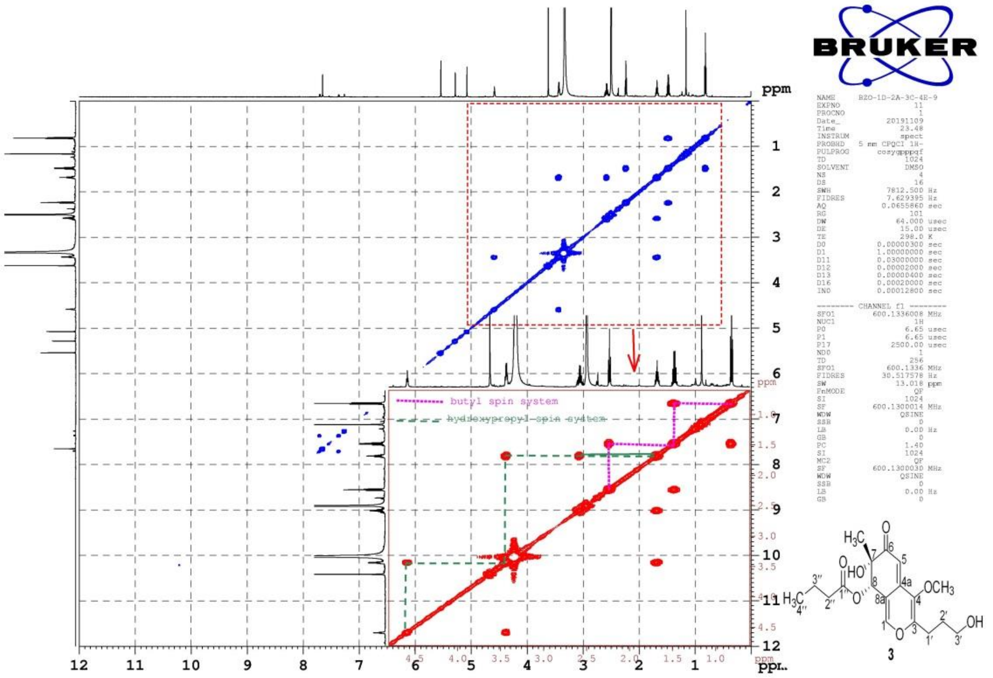

Figure S23. The ^{1}H-^{1}H COSY spectrum of compound **3** in DMSO-d_6

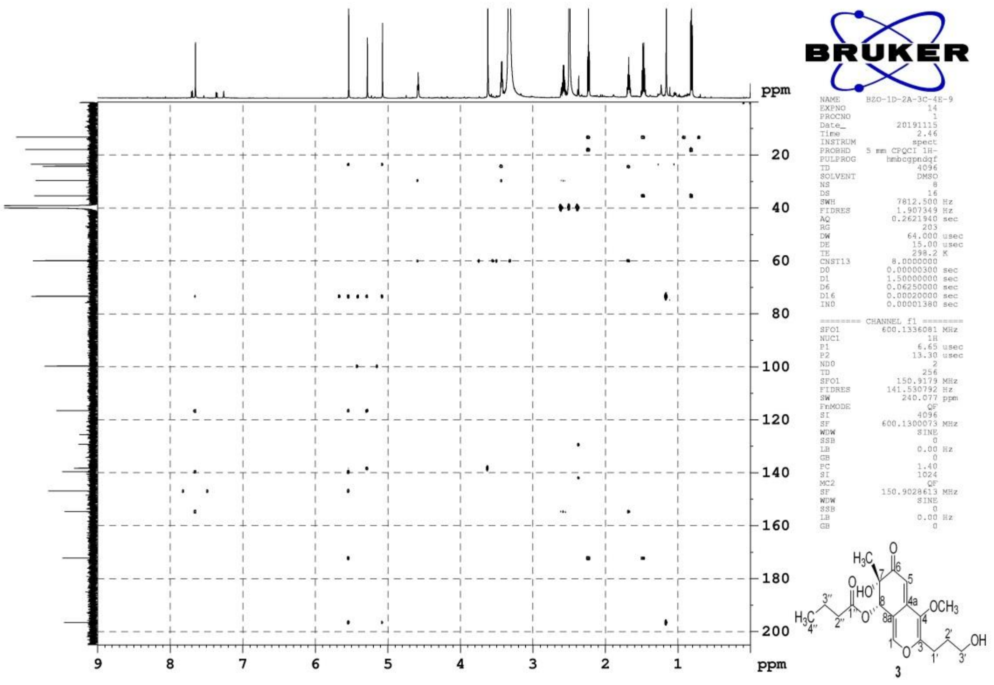

Figure S24. The HMBC spectrum of compound **3** in DMSO-d_6

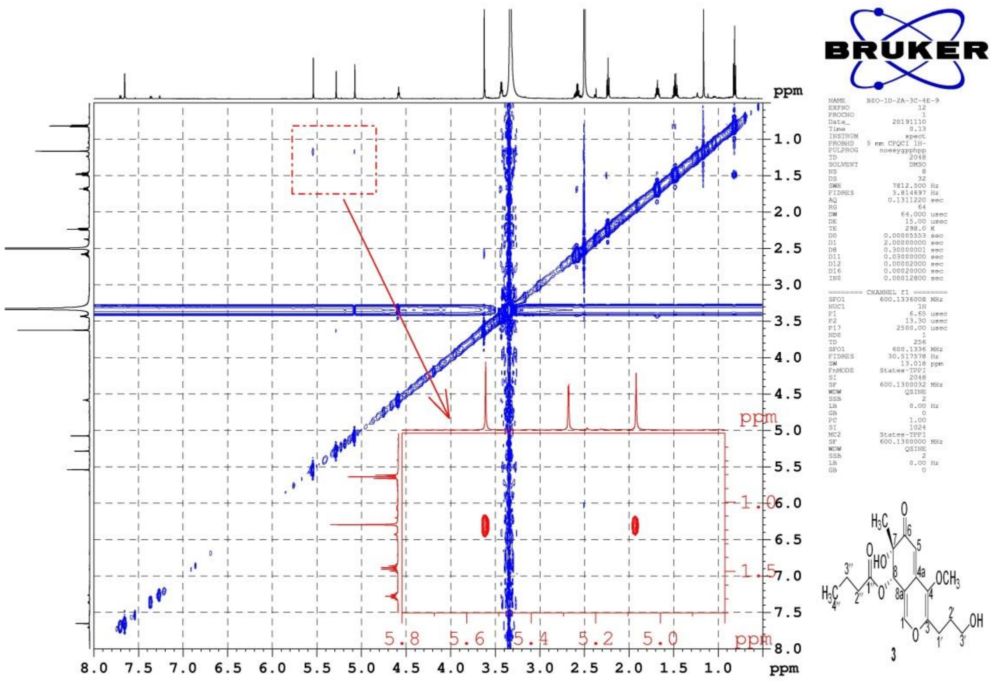

Figure S25. The NOESY spectrum of compound **3** in DMSO-d_6
